# Control of Light Transmission in a Plasmonic Liquid Metacrystal

**DOI:** 10.3390/nano11020346

**Published:** 2021-02-01

**Authors:** Alexander Zharov, Zacharias Viskadourakis, George Kenanakis, Vanessa Fierro, Alain Celzard

**Affiliations:** 1 Université de Lorraine, CNRS, IJL, F-88000 Epinal, France; alexander.zharov@univ-lorraine.fr (A.Z.); vanessa.fierro@univ-lorraine.fr (V.F.); 2 Institute for Physics of Microstructures, Russian Academy of Sciences, 603950 Nizhny Novgorod, Russia; 3 Institute of Electronic Structure and Laser (IESL), Foundation for Research & Technology—Hellas (FORTH), N. Plastira 100, Vasilika Vouton, GR-700 13 Heraklion, Crete, Greece; zach@iesl.forth.gr (Z.V.); gkenanak@iesl.forth.gr (G.K.)

**Keywords:** liquid metamaterials, liquid metacrystals, plasmonic metamaterials

## Abstract

In this study, we experimentally demonstrated the control of light transmission through a slab of plasmonic liquid metacrystal by an external electric field. By applying the external static field, we were able to induce macroscopic anisotropy, which caused the polarization-dependent suppression of transmission at resonant frequencies. Such behavior indicates the selective plasmon excitation governed by the orientation of the meta-atoms with respect to the polarization of the electromagnetic wave. The problem of light transmission through a plasmonic liquid metacrystal was analyzed theoretically from first principles, and the obtained results were compared with the experimental data.

## 1. Introduction

Metamaterials offer the possibility to control optic fields at micro- or nanoscale, which makes them an invaluable tool in modern photonics. In recent years, various types of metamaterials have been studied both theoretically and experimentally. These include left-handed materials [1,2,3,4,5], non-linear metamaterials [6,7], hyperbolic materials [8,9,10], near-zero-index metamaterials [11,12] and liquid metamaterials [13]. The specially designed properties, which are absent in natural materials, have allowed metamaterials to be used in applications such as invisibility cloaking [14,15], superlensing [16,17], transformation optics [18,19], and enhancement of chirality and optical activity [20,21,22,23,24].

Liquid metamaterials have received special attention because of their high tunability and reconfigurability. In particular, nanocluster plasmonic metafluids and negative index metafluids have been studied theoretically [25,26], while colloidal metamaterials and hybrid liquid crystal-based colloids have been studied experimentally [27,28,29,30,31].

A particular type of liquid metamaterials called liquid metacrystals (LMC) was introduced in [32,33,34]. An LMC is an array of anisotropic subwavelength particles suspended in a viscous liquid. Like a regular liquid crystal, such a medium has no spatial order, while the orientational order can be induced by an external static electric field, leading to the appearance of macroscopic anisotropy. At the same time, the meta-atoms can be designed for resonant response at certain frequencies. An LMC specifically tuned for THz radiation has been demonstrated experimentally in [35].

In this study, we demonstrate experimentally the control of electromagnetic wave (EMW) transmission through a slab of LMC by an external electric field. The LMC medium was composed of plasmonic gold nanorods suspended in deionized water and stabilized by cetyltrimethylammonium bromide used as capping agent. The nanorods were oriented by a static electric field, which provided macroscopic anisotropy of the medium and suppressed the EMW transmission at certain frequencies and polarizations.

## 2. Theory

The main property of an LMC is the strong macroscopic anisotropy induced by an external static electric field, which comes from the anisotropy of each meta-atom. Namely, in the absence of an external field, meta-atoms are oriented randomly, and their macroscopic ensemble is therefore isotropic. However, a static electric field orients the meta-atoms in the same direction, resulting in the appearance of an average anisotropy of such a medium as a whole. To detect such behavior, we study the influence of the static electric field on the transmission of two orthogonal linear polarizations through an LMC film. Therefore, we must first find the transmission coefficients for this system.

Let us consider an LMC at temperature *T* in a static electric field $E=z_{0}\;E_{0}$, where $z_{0}$ is the unit vector along the *z*-axis. The angular distribution of the meta-atoms can be described by the Boltzmann distribution function (see, e.g., [36]):

(1) $$f\left(\theta \right)=A\exp\left[\frac{\delta \alpha^{0}E_{0}^{2}\cos^{2}\theta }{k_{B}T}\right],$$

where θ is the angle between the *z*-axis and anisotropy axis of the meta-atoms, $\delta \alpha^{0}=\alpha_{\|}^{0}-\alpha_{⊥}^{0}$ is the difference between the longitudinal $\alpha_{\|}^{0}$ and transverse $\alpha_{⊥}^{0}$ static polarizabilities of a meta-atom, and $k_{B}$ is the Boltzmann constant. The constant *A* is defined by the normalization condition $\int_{0}^{2\pi }d\phi \int_{0}^{\pi }d\theta \;f\left(\theta \right)\sin\theta =N$, where *N* is the concentration of the particles.

**Figure 1:**
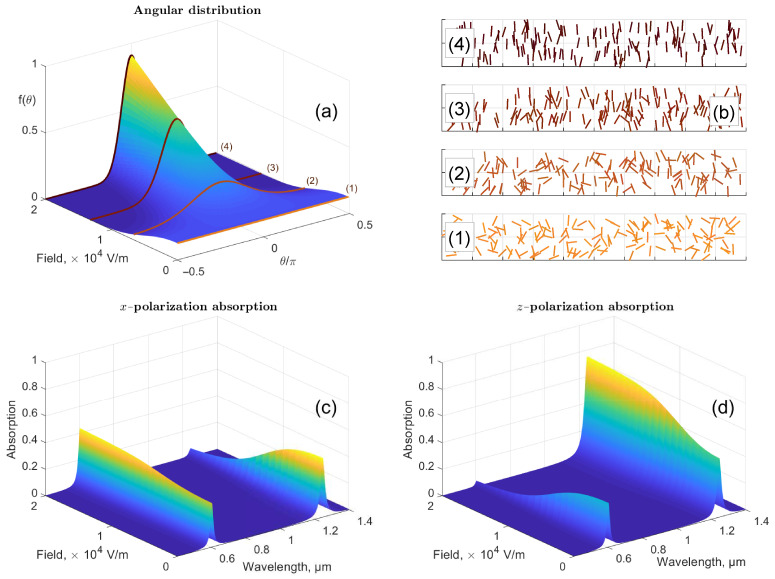
Influence of the external electric field on the characteristics of the particle suspension. (**a**) Angular distribution function as a function of the external electric field. For easier visualization, the definition domain of $\theta \in \left[0,\pi \right]$ was translated into $\left[-\pi /2,\pi /2\right]$. (**b**) Examples of particle distributions at four different values of external field, E=0.0 (1), 6.6 (2), 13.2 (3), and 20.0 (4) kV/m. Indices (1)–(4) correspond to those in (**a**); the corresponding distribution functions were used to simulate the random distributions shown in (**b**). Absorption of an EMW in an LMC slab as a function of frequency and external electric field for: (**c**) *x*-polarization, and (**d**) *z*-polarization. The plots were calculated for ellipsoidal 10 nm × 80 nm gold nanorods (aspect ratio of 8) in water at a concentration of $10^{13}$ ${\mathrm{mL}}^{-1}$.

The evolution of the angular distribution as a function of the external field is shown in Figure 1a,b. When no external field is applied, the particles are distributed evenly, and there is no preferred orientation direction (see Figure 1(a,b(1))). On the other hand, with the growth of the external field, the particles tend to orient along it. As a result, a peak near θ=0 forms in the distribution function, indicating the appearance of the preferred orientation direction (see Figure 1(a,b(2)–(4))).

To find the macroscopic electromagnetic properties of the LMC, the corresponding values have to be averaged over the distribution given by Equation (1), [36]. For instance, the averaged *x*- and *z*-dynamic polarizabilities of the LMC can be found as:

$$\begin{array}{c} {\tilde{\alpha }}_{xx}\left(\omega \right)=4\pi \nu_{0}\epsilon_{0}\left(\frac{\alpha_{⊥}\left(\omega \right)+\alpha_{\|}\left(\omega \right)}{2}-\right. \end{array}$$

(2) $$\begin{array}{c} \left.\frac{\alpha_{\|}\left(\omega \right)-\alpha_{⊥}\left(\omega \right)}{4}\left(\frac{e^{\kappa^{2}}}{\zeta \left(\kappa \right)\kappa }-\frac{1}{\kappa^{2}}\right)\right),\\ {\tilde{\alpha }}_{zz}\left(\omega \right)=4\pi \nu_{0}\epsilon_{0}\left(\alpha_{⊥}\left(\omega \right)+\right. \end{array}$$

(3) $$\begin{array}{c} \left.\frac{\alpha_{\|}\left(\omega \right)-\alpha_{⊥}\left(\omega \right)}{2}\left(\frac{e^{\kappa^{2}}}{\zeta \left(\kappa \right)\kappa }-\frac{1}{\kappa^{2}}\right)\right), \end{array}$$

where $\alpha_{\|}\left(\omega \right)$ and $\alpha_{⊥}\left(\omega \right)$ are the longitudinal and transverse dynamic polarizabilities of a meta-atom, ω is the angular frequency, $\nu_{0}=NV$ is the volume fraction of meta-atoms, where *N* and *V* are the concentration and volume of meta-atoms, respectively, $\epsilon_{0}$ is the permittivity of vacuum, $\kappa =\sqrt{\delta \alpha^{0}E_{0}^{2}/k_{B}T}$ is the dimensionless static electric field, and $\zeta \left(x\right)=\int_{0}^{x}\exp\left(x^{2}\right)dx$. Thus, the corresponding components of the permittivity tensor can be estimated as [37]:

(4) $$\epsilon_{xx\left(zz\right)}\left(\omega \right)=\epsilon_{l}\epsilon_{0}+{\tilde{\alpha }}_{xx\left(zz\right)}\left(\omega \right),$$

where $\epsilon_{l}$ is the relative permittivity of the surrounding fluid. According to Equation (4), the correction to the permittivity tensor due to the presence of meta-atoms ${\tilde{\alpha }}_{xx\left(zz\right)}\left(\omega \right)$ is proportional to their concentration. As a result, the reflection and transmission coefficients also depend on the meta-atom concentration.

The reflection *R* and transmission *T* coefficients for the *x*- and *z*-polarized beams normally incident on an LMC slab of thickness *d* can be calculated as:

(5) $$R_{x\left(z\right)}=R_{0x\left(z\right)}\frac{1-\exp\left(-2ik_{0}n_{x\left(z\right)}d\right)}{1-R_{0x\left(z\right)}^{2}\exp\left(-2ik_{0}n_{x\left(z\right)}d\right)}$$

and

(6) $$T_{x\left(z\right)}=1-\frac{R_{x\left(z\right)}}{R_{0x\left(z\right)}},$$

where $R_{0x\left(z\right)}=\left(n_{x\left(z\right)}-1\right)/\left(n_{x\left(z\right)}+1\right)$ with $n_{x\left(z\right)}=\sqrt{\epsilon_{xx\left(zz\right)}\left(\omega \right)}$, and $k_{0}=\omega /c$ where *c* is the speed of light.

The absorption coefficients, $A_{x\left(z\right)}=1-{\left|T_{x\left(z\right)}\right|}^{2}-{\left|R_{x\left(z\right)}\right|}^{2}$, as functions of frequency and external electric field for *x*- and *z*-polarizations, are shown in Figure 1c,d. It is clear that without external field, the absorption coefficients are similar for both polarizations. On the other hand, the non-zero external field suppresses the long-wavelength (longitudinal) and enhances the short-wavelength (transverse) plasmon peaks for the *x*-polarization, and, on the contrary, enhances the longitudinal and suppresses the transverse plasmon peaks for the *z*-polarization.

In the analysis above, it was assumed that all particles have exactly the same shape and, therefore, exactly the same longitudinal and transverse polarizabilities. However, as size dispersion is always present, its influence on the LMC properties should also be analyzed.

To validate this hypothesis, we further consider prolate ellipsoidal nanoparticles. Their polarizabilities can be calculated as follows [38]:

(7) $$\begin{array}{c} \alpha_{\|}\left(\omega ,\;n\right)=\frac{a^{2}b}{3}\frac{\epsilon \left(\omega \right)-\epsilon_{l}}{\epsilon_{l}+1/2\left(\epsilon \left(\omega \right)-\epsilon_{l}\right)\left(1-n\right)}, \end{array}$$

(8) $$\begin{array}{c} \alpha_{⊥}\left(\omega ,n\right)=\frac{a^{2}b}{3}\frac{\epsilon \left(\omega \right)-\epsilon_{l}}{\epsilon_{l}+\left(\epsilon \left(\omega \right)-\epsilon_{l}\right)n}, \end{array}$$

where *a* and *b* are the ellipsoid semi-axes, $\epsilon \left(\omega \right)$ is the frequency-dependent permittivity of the particle material, $\epsilon_{l}$ is the permittivity of the surrounding liquid, and *n* is the so-called depolarization factor, which is defined as

(9) $$n=\frac{1}{2}{\left(\frac{a}{b}\right)}^{2}\int_{0}^{\infty }\frac{d\xi }{{\left(1+\xi \right)}^{3/2}\left(\xi +{\left(a/b\right)}^{2}\right)}.$$

**Figure 2:**
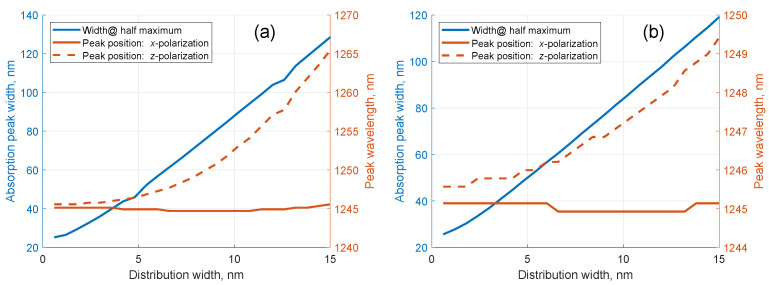
Dependence of the width at half maximum and the positions of the longitudinal plasmon absorption peak for *x*- and *z*-polarizations on the width of the particle size distribution for rectangular (**a**) and Gaussian (**b**) distributions.

The depolarization factor depends only on the aspect ratio a/b and, in particular, defines the measure of the splitting between the longitudinal and transverse plasmons in metallic particles. Thus, the size dispersion of the particles can be simulated by adding the *n*-dependent part into the distribution function, Equation (1), and keeping the normalization such that the number of particles of a certain size is preserved. Namely,

(10) $$f\left(\theta ,\;n\right)=A^{\prime }\exp\left[\frac{\delta \alpha^{0}E_{0}^{2}\cos^{2}\theta }{k_{B}T}\right]\phi \left(n\right),$$

where $\phi \left(n\right)$ is the depolarization factor distribution and the normalization constant $A^{\prime }$ is defined such that $\int d\theta \sin\theta f\left(\theta ,n\right)=N_{p}\left(n\right)$, where $N_{p}\left(n\right)$ is the partial concentration of particles with the depolarization factor *n*, $\int dnN_{p}\left(n\right)=N$. To find the macroscopic characteristics, again, the corresponding values have to be averaged over the distribution; however, the dependence of the depolarization factor on the polarizability must be taken into account. Figure 2 shows the width at half maximum and the position of the maxima of the longitudinal plasmon absorption for *x*- and *z*-polarizations as a function of the width of the particle size distribution. In Figure 2a, the size distribution is assumed rectangular, while in Figure 2b the distribution is assumed Gaussian. It can be seen that, along with the evident widening of the absorption peak, a slight red shift of the *z*-polarized absorption takes place. This phenomenon can be understood in view of the fact that the particles with a higher aspect ratio (and a lower longitudinal plasmon frequency) are easier to orient, and thus they contribute more to the *z*-polarization absorption than shorter particles when the external field is applied.

## 3. Experiment

**Figure 3:**
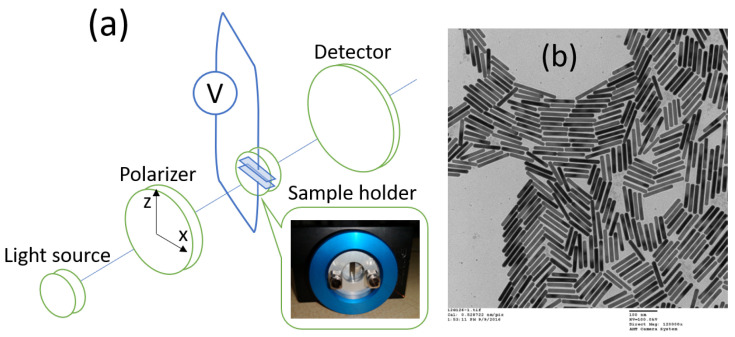
(**a**) Schematic representation of the experimental setup. (**b**) Electron microscope image of gold nanorods (provided by Nanopartz Inc., Loveland, CO, USA).

The FT-IR (transmission) measurements were performed using a Bruker Vertex 70v FT-IR vacuum spectrometer (Bruker Corporation, Billerica, MA, USA), using a quartz beam-splitter and a room temperature silicon diode detector, in the wavelength range of 0.45–1.4 μm. A demountable liquid cell (obtained from PIKE Technologies, Fitchburg, WI, USA), with two CaF_2_ windows (3 mm thick) separated by a polytetrafluoroethylene (PTFE) spacer ring (with an inner diameter of 10 mm, and a thickness of 40–50 microns) was used in order to measure the transmission of the liquid metacrystal. In addition, two flat semi-circle-type metallic electrodes (10 mm diameter; 25–30 μm thickness) were fitted in the above-mentioned PTFE spacer ring, in order to provide a static electric field varying from 0 to 60 V, leaving a measurement area (gap) of 3 mm × 10 mm as seen in Figure 3a. Finally, a rotated linear polarizer (transmission >90%; polarization degree >500:1) was used to provide two different linear polarizations along either *x*- or *z*-axis.

The liquid metacrystal sample, based on a suspension of gold nanorods (Nanopartz Inc., Loveland, CO, USA; size: 10 nm × 81 nm; concentration: $10^{13}$ ${\mathrm{mL}}^{-1}$) in deionized water, was placed in the liquid cell described above, and transmission measurements were performed for two different linear polarizations, under a DC voltage of 0–60 V. In each measurement, interferograms were collected at a resolution of 8 ${\mathrm{cm}}^{-1}$ (10 scans), apodized with a Blackman–Harris function, and Fourier-transformed with two levels of zero filling to yield spectra encoded at 2 ${\mathrm{cm}}^{-1}$ intervals. Prior to scanning the samples, a background measurement was recorded using the empty demountable liquid cell described above, and each sample spectrum was obtained by automatic subtraction of it.

At this point, it should be noted that the liquid sample was sonicated for 5 min to avoid precipitation and to redisperse the nanorods before each experiment; the electron microscope image of the nanorods is shown in Figure 3b. Furthermore, in order to check the solubility of CaF_2_ and the stability of our setup against leakages, etc., we checked it using deionized water, before suspending the gold nanorods, under a DC voltage of 0–60 V, and neither instability issues nor electrical noise were detected.

## 4. Results and Discussion

**Figure 4:**
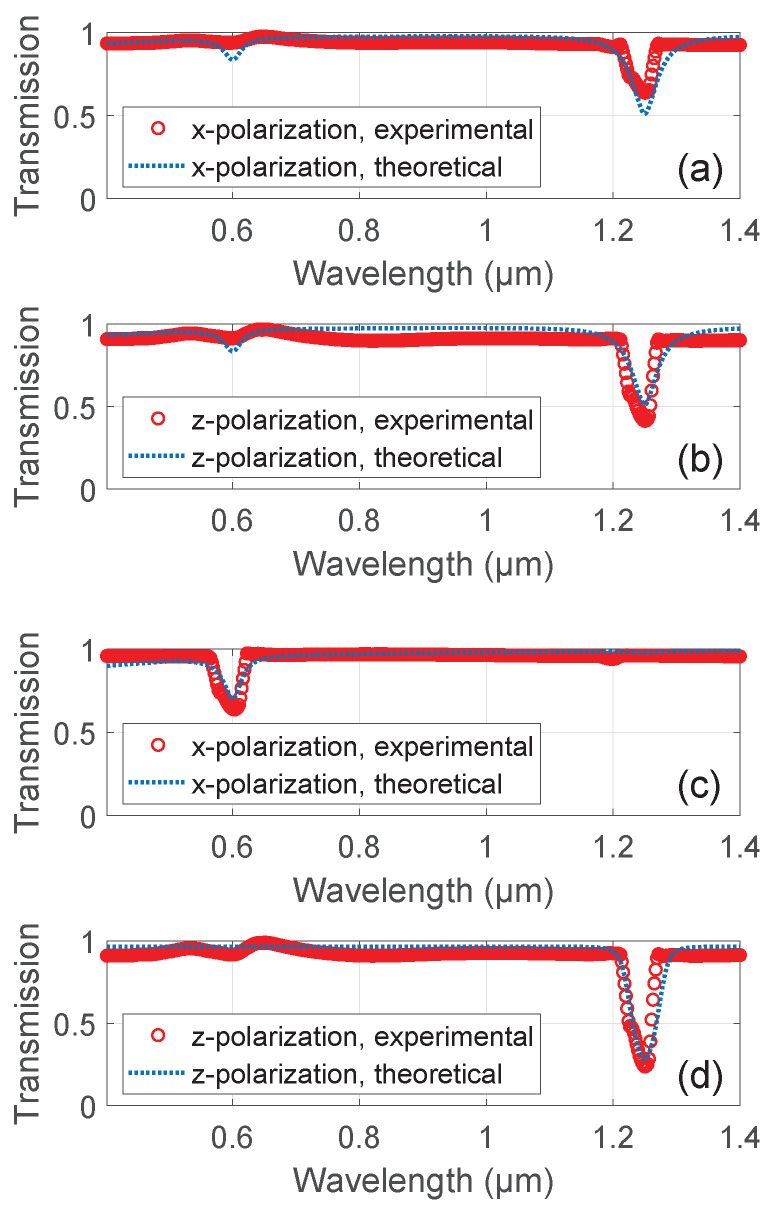
Comparison of experimental (red circles) and theoretical (blue dots) transmissions through a slab of LMC as a function of wavelength at (**a**) 0 V, *x*-polarization, (**b**) 0 V, *z*-polarization, (**c**) 60 V, *x*-polarization, and (**d**) 60 V, *z*-polarization.

Figure 4 shows the wavelength dependence of the transmission of two perpendicular linear polarizations at two values of voltage, 0 and 60 V. The experimentally observed transmission is compared with the theoretical calculations based on Equations (5) and (6). The high-frequency permittivity of gold was obtained from the refractive index database [39]. It can be seen that without external electric field (0 V, Figure 4a,b), the longitudinal plasmon peaks for *x*- and *z*-polarizations at 1250 nm are almost similar. The transverse plasmon peaks at 600 nm are also almost similar. However, these peaks are suppressed for both polarizations with respect to the theoretical calculation. On the other hand, in the presence of the orientational field (60 V, Figure 4c,d), for *z*-polarization, the longitudinal plasmon is enhanced, and the transverse plasmon is suppressed, while for *x*-polarization, the longitudinal plasmon is suppressed, and the transverse plasmon is enhanced.

Such a behavior is substantially consistent with the theoretical prediction of Equations (5) and (6). Indeed, in the absence of external field, the particles are randomly oriented, and the absorption is independent of polarization. A slight difference between the experimental *x*- and *z*-polarization absorptions (Figure 4a,b, respectively) may be attributed to the residual particle clustering. On the other hand, when the particles are oriented along the *z*-axis, the *z*-polarization effectively excites the longitudinal plasmon, and the *x*-polarization excites the transverse one. In turn, this selective plasmon excitation leads to the enhanced absorption of the selected polarization at the corresponding plasmon frequency.

## 5. Conclusions

In this paper, we have experimentally demonstrated the controllable EMW transmission through a plasmonic LMC in the optical frequency band. The polarization-dependent modulation of transmission governed by the applied static electric field has been observed. The selective plasmon excitation controlled by the radiation polarization and the static electric field has been evidenced. The results of the theoretical analysis were found to be in good agreement with the experimental data.

## Data Availability

All data reported here can be made available on request.
